# IS IT LONELY AT THE TOP? BIOPSYCHOSOCIAL VARIANTS OF LONELINESS AMONG CENTENARIANS

**DOI:** 10.1093/geroni/igz038.148

**Published:** 2019-11-08

**Authors:** Alex J Bishop, Oscar Riberio

**Affiliations:** 1 Oklahoma State University, Stillwater, Oklahoma, United States; 2 University of Aveiro - Department of Education and Psychology | Center for Health Technology and Services Research (CINTESIS.UA), Aveiro, Portugal

## Abstract

There is a growing body of evidence supporting the detrimental impact of loneliness on biological, psychological, and social functioning. Loneliness has been cited to contribute to social isolation, mental health disorders, and premature death in old age. In turn, the prevention of loneliness has emerged as a priority area in geriatric and gerontology research, practice, and policy. However, determination of whether persons living 100 or more years feel lonely or socially disconnected has remained limited within centenarian research. Such research has historically fostered translation of insights and secrets for living long and aging well. Centenarians represent persons who have managed to survive, delay, or escape varying biopsychosocial losses that might otherwise deteriorate emotional health, exacerbate feelings of isolation, and limit human longevity potentials. Guided by a biopsychosocial framework, this symposium will consider biological, psychological, and social variants that contribute to risk as well as resilience in loneliness in very old age. Of particular interest is the advancement of evidence-based research exposing the interplay between loneliness and nutritional health, impact of lifelong childlessness on feelings of solitude, role of personality traits and the expression of loneliness, and the intersection between active religious engagement and loneliness. Biopsychosocial attributes that reduce the threat of social isolation and loneliness, as well as improve emotional well-being in human longevity will be further discussed. Implications relevant for geriatric counseling and wellness programming for old-old adults will be highlighted.

